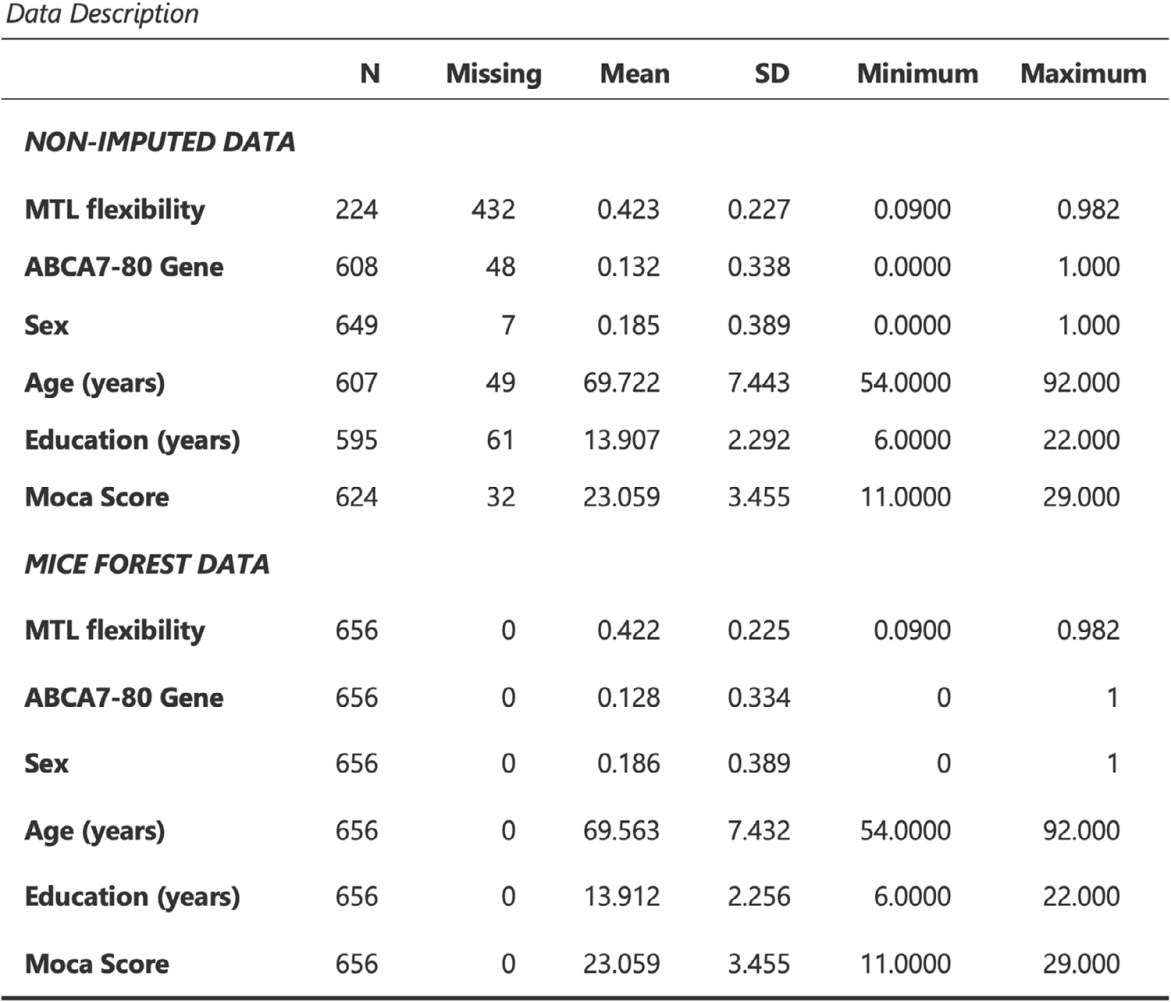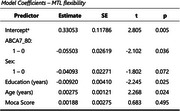# Medial Temporal Lobe Flexibility as an Early Marker of Alzheimer’s Risk in African Americans with ABCA7‐80 Variant: Application to Multivariate Imputation by Chained Equations using Random Forest

**DOI:** 10.1002/alz70861_108696

**Published:** 2025-12-23

**Authors:** Rutvik Deshpande, Soodeh Moallemian, Abolfazl Saghafi, Miray Budak, Bernadette A. Fausto, Mark A. Gluck

**Affiliations:** ^1^ Center for Molecular & Behavioral Neuroscience, Rutgers University–Newark, Newark, NJ USA; ^2^ Rutgers University–Newark, Newark, NJ USA; ^3^ Sait Joseph's University, Philadelphia, PA USA

## Abstract

**Background:**

Alzheimer’s disease (AD) is characterized by progressive neurodegeneration and cognitive decline. Medial Temporal Lobe (MTL) Flexibility, measured from dynamic functional connectivity in resting‐state fMRI, may serve as a biomarker for Mild Cognitive Impairment (MCI) and AD. The ABCA7‐80 variant is associated with increased dementia risk, particularly among African Americans; however, few studies have examined its relationship with MTL Flexibility. Furthermore, missing data remains a pervasive challenge in AD research, often driven by participant burden and health factors. This study investigates the association between ABCA7‐80 and MTL Flexibility, using Multivariate Imputation by Chained Equations Forest (MICEforest) to address high amount of missing data.

**Method:**

656 participants were included, enrolled in the Pathways to Healthy Aging in African Americans study. Participants underwent blood draws, MRI scans, and Montreal Cognitive Assessment (MoCA). We first performed partial correlation between ABCA7‐80 gene and MTL Flexibility, adjusting for age, sex, education on the 224 participants (Mean*_age_* = 69.7 ± 7.2 years) with MTL flexibility score (pairwise deletion technique). Then a linear regression model was fitted on the data to predict MTL flexibility using ABAC7‐80. Same analyses were used to test the data after MICEforest imputation on the full sample (n = 656; Mean*_age_* = 69.5 ± 7.4 years).

**Result:**

In the pairwise deleted dataset, almost significant association was found between MTL Flexibility and ABCA7‐80 (r = ‐0.141, *p* > 0.058) after adjusting for age, sex, education and MoCA score. After imputing the missing data, ABCA7‐80 high‐risk allele carriers showed significantly lower MTL Flexibility (r = ‐0.082, *p* < 0.05). Our regression analysis on the imputed data reveals that ABCA7‐80 can predict the MTL flexibility after controlling for age, sex, education and cognition (R² = 0.026, *p* = 0.003).

**Conclusion:**

Our findings reinforce the role of ABCA7‐80 as a genetic risk factor for AD and suggest reduced MTL Flexibility as an early biomarker of vulnerability. Notably, MICEforest imputation empowered our analyses revealing statistically significant correlation and a linear regression model between MTL Flexibility and ABCA7‐80 gene. This highlights the importance of robust imputation strategies for maximizing the utility of neuroimaging data in aging and dementia research.